# Investigating how the GRADE Evidence to Decision (EtD) framework is used in Clinical Guidelines: a scoping review protocol

**DOI:** 10.12688/hrbopenres.13757.1

**Published:** 2023-09-13

**Authors:** Ruairí Murray, Melissa Sharp, Adriana Razidan, Ben Hibbitts, Máirín Ryan, Kamal Mahtani, Rosarie Lynch, Susan Smith, Michelle O'Neill, Holger Schünemann, Pablo Alonso-Coello, Zachary Munn, Barbara Clyne

**Affiliations:** 1 Health Technology Assessment, Health Information and Quality Authority, Dublin, D07 E98Y, Ireland; 2 Department of Public Health & Epidemiology, School of Population Health, RCSI University of Medicine and Health Sciences, Dublin, Ireland; 3 School of Pharmacy and Biomolecular Sciences (PBS), RCSI University of Medicine and Health Sciences, Dublin, Ireland; 4 Department of Pharmacology & Therapeutics, Trinity College Dublin, Trinity Health Sciences, Dublin, D02 PN40, Ireland; 5 Nuffield Department of Primary Care Health Sciences, University of Oxford, Oxford, England, UK; 6 Department of health, Clinical Effectiveness and Antimicrobial Resistance Unit, National Patient Safety Office, Dublin, Ireland; 7 Department of Public Health and Primary Care, School of Medicine, Trinity College Dublin, Dublin, D02 PN40, Ireland; 8 Department of Health Research Methods, Evidence, and Impact McMaster University, Ontario, Canada; 9 CIBER of Epidemiology and Public Health, Iberoamerican Cochrane Center-Servicio de Epidemiología Clínica y Salud Pública, Biomedical Research Institute, Barcelona, Spain; 10 Faculty of Health and Medical Sciences, JBI Adelaide GRADE Centre, University of Adelaide, Adelaide, Australia

**Keywords:** GRADE, guidelines, Decision-making, methodology, scoping review

## Abstract

**Introduction:** The Grading of Recommendations, Assessment, Development and Evaluation (GRADE) evidence to decision (EtD) framework provides a structured and transparent approach for clinical guideline developers to use when formulating recommendations. Understanding how stakeholders use the EtD framework will inform how best to provide future training and support. This scoping review objective is to identify the key characteristics of how the GRADE EtD framework is used and identify studies on perception of use by those involved in developing clinical guidelines.

**Methods:** JBI methodology for scoping reviews will be followed. This scoping review will consider both peer review published literature and grey literature. This will include empirical studies on the use of EtDs (including both quantitative, qualitative, and mixed methods primary research articles) and discussion papers/ commentaries on the experience of using the EtD. It will also include a random sample of publicly available populated EtDs identified from databases and repositories of GRADE guidelines. The search strategy will aim to locate both published and unpublished documents. First, we will conduct an exploratory search of MEDLINE and Embase (Elsevier), supplemented with citation analysis of included articles. Populated EtDs will be identified through searches of databases and repositories of GRADE guidelines. Two researchers will independently screen, select, and extract identified documents. Data will be presented in tables and summarized descriptively.

**Conclusion:** This scoping review will identify the key characteristics of how the GRADE EtD framework is currently being used in clinical guidelines. Review findings can be used to inform future guidance and requirements for using GRADE EtD, as well as training on how to consider the criteria in developing recommendations. Results will be disseminated through publications in peer – reviewed journals and conference presentations. We will present our findings to relevant stakeholders via the networks of the co-author team at a one-day workshop.

## Introduction

Clinical guidelines are considered a key foundation for quality improvement in health care ^1^. They are systematically developed statements to assist practitioner and patient decisions about appropriate health care for specific clinical circumstances ^1^. The development of guidelines is intended to organize and provide the best available evidence to support clinical decision making in order to improve quality of care, patient outcomes, and cost-effectiveness.

Ideally, within a guideline, each recommendation will be presented with a rating of both its strength and the certainty of the underlying evidence. The guideline development groups (GDGs) charged with developing these recommendations generally comprise a mix of key clinical and non-clinical stakeholders. These diverse groups bring a plurality of experiences, perspectives, and backgrounds to the discussions informing evidence-based recommendations. However, this complexity can produce different interpretations of the same evidence by different stakeholders, as patients and physicians may process, interpret, and respond to various types of uncertainty inherent in clinical decisions in different ways ^2– 4^. Therefore, a systematic approach that helps groups consider all the relevant factors can facilitate a more structured and explicit process when developing recommendations ^5^.

The Grading of Recommendations, Assessment, Development and Evaluation (GRADE) approach, used internationally, presents a methodologically rigorous and transparent system for making judgments about the certainty of evidence and strength of recommendations ^6^. The GRADE Evidence to Decision (EtD) framework ( Table 1) was developed as a systematic and transparent way for guideline developers and decision makers to structure the process of moving from evidence to decisions, using pre-specified criteria ^5^. These nine criteria (as outlined in Table 1) aim to ensure that important factors that determine a decision are considered in the decision-making process and a number of tailorable GRADE-EtD framework templates and guidance documents have been developed ^5, 7, 8^. More than 100 organisations globally have adopted the principles of the GRADE system ^5^, many of which include detail on using GRADE EtD in their methodological guidance documents ^9^. Although GRADE has been widely endorsed, its use may be inconsistent with GRADE guidance and there is variation across organisations in terms of how the EtD should be adopted and applied ^9, 10^. The level of supporting evidence provided in published EtDs for core criteria such as resources required and cost effectiveness ^11^, and equity ^12^ has also been shown to vary.

**Table 1.** Criteria for an EtD framework.

Criterion	Question prompts
Problem priority	• Is the problem a priority?
Benefits and harms	• How substantial are the desirable anticipated effects? • How substantial are the undesirable anticipated effects?
Certainty of the evidence	• What is the overall certainty of the evidence of effects?
Outcome importance	• Is there important uncertainty about or variability in how much people value the main outcomes?
Balance	• Do the desirable effects outweigh the undesirable effects?
Resource use	• How large are the resource requirements? • What is the certainty of the evidence of resource requirements?
Equity	• What would be the impact on health equity?
Acceptability	• Is the intervention/option acceptable to key stakeholders?
Feasibility	• Is the intervention feasible to implement?

The use of an EtD framework is associated with guidelines of better quality, and more credible and transparent recommendations ^13^. It has been reported to help in structuring a complex process through relatively simple steps in an explicit and transparent way ^14^, managing discussions, keeping guideline panels on track, and dealing with disagreements ^15^. However, to take full advantage of the EtD framework, it is necessary to be familiar with the GRADE approach and the framework itself. Therefore, for new users, prior training may be helpful for successful use ^14^. While numerous guidance documents have been developed ^5, 7, 8^, emerging evidence has highlighted that there is variation in terms of how EtDs are being adopted and applied by organisations ^9, 10^. The first study assessing the use of GRADE's EtD framework during real-time guideline development using panel discussions found that the proportion of time dedicated to certain framework criteria (as outlined in Table 1) is greater than others (e.g. research evidence 53% *versus* equity 2%) ^16^. Further studies have indicated that the level of supporting evidence provided in published EtDs for criteria such as resources required and cost effectiveness ^11^, and equity ^12^ has also been shown to vary. Factors contributing to this disparity were unclear.

Given the widespread use and endorsement, of the GRADE EtD approach and its potential to increase the quality of guidelines ^13^, understanding how they are currently utilised and perceived may identify areas of variation and the contributing factors, as well as informing how best to provide training and support - an important facilitator of engagement, particularly for patients ^17^. Therefore, this scoping review aims to identify the key characteristics of how the GRADE EtD is used and identify studies on perception of use by clinical guideline developers.

### Review question

How do guideline development groups apply, populate, and perceive the GRADE EtD when developing recommendations in clinical guidelines?

Specific questions:

- How is the GRADE EtD being used in GDG meetings in terms of the approaches to completing the EtD, guiding panel discussions and reaching consensus?
- Are there studies on how the GRADE EtD is perceived by various stakeholders involved in GDGs?
- How are the sections of the GRADE EtDs populated in terms of the research evidence and additional considerations supplied/cited to support developing recommendations?

## Methods

The proposed scoping review will be conducted in accordance with the JBI methodology for scoping reviews ^18^ and reported in line with the Preferred Reporting Items for Systematic Reviews and Meta-analyses (PRISMA) extension for scoping reviews ^19^. This scoping review aligns to the indication for conducting a scoping review in that it aims to identify key characteristics or factors related to a concept, including those related to methodological research ^20^. The protocol has been registered with Open Science Framework ^21^.

### Eligibility criteria

***Participants***. The focus of this review is on how GDGs apply the GRADE EtD, therefore participants in included studies will be guideline panel members, including but not limited to healthcare professionals, managers, methodologists, and patient representatives.

***Concept***. The concept of interest is how GDGs apply and perceive the GRADE EtD. This includes the approach to using the framework within panel meetings, how the framework is populated and with what sources, how it is presented and communicated, and the perspectives of those using the framework.

***Context***. This review will be limited to the inclusion of EtDs used in clinical guidelines. Guidelines can target any clinical area and can be at a local, national, regional, or global level. We will include guideline EtDs (provided in English) and evaluations. For guidelines that have been updated, only the most recent version will be included.

***Types of documents***. This scoping review will consider both peer reviewed published literature and grey literature. This will include empirical studies on the use of EtDs (including quantitative, qualitative, and mixed methods primary research articles) and discussion papers/ commentaries on the experience of using the EtD. Manuals and guidance documents on how to use the EtD, or articles describing its development or structure will not be included. Articles providing detailed descriptions of guidelines and the methodology to develop them will also be excluded. There will be no exclusions based on language or publication status (i.e., published, in press, in progress, or pre-print) for peer reviewed literature. For documents in languages other than English, Google Translate will be used, supplemented by interpretation by a native speaker where possible. Given the complexity of guideline EtDs and the potential for misinterpreting context, only EtDs produced in English will be included.

It will also include a random sample of populated EtDs (n=200) that have been made publicly available identified from databases and repositories of GRADE guidelines listed below. Given the number of published guidelines and EtDs published, it would not be feasible to include them all, therefore we have chosen an approach to restricting the numbers, as previous analyses have also done ^13^. These guidelines can be created by any group but do need to have used a version of the GRADE EtD.

### Search strategy

The search strategy, developed with an expert health librarian (PM), will aim to locate both published and unpublished documents. An initial exploratory search of Medline was undertaken to identify key articles on the topic. The terminology used within the articles was analysed and used to develop a full search strategy ( Table 2) for both Medline and Embase (Elsevier), conducted April 2023. The search will be supplemented by a citation analysis of the seminal EtD papers.

**Table 2.** Database search: MEDLINE (Ovid).

Term
1. (Guideline$ adj1 development).mp.
2. Group decision making.mp
3. (guideline adj2 panel).mp.
4. guideline group$.mp.
5. 1 or 2 or 3 or 4
6. Evidence to decision framework.mp.
7. ((GRADE adj1 EtD) or (GRADE adj2 iEdT)).mp.
8. (EtD framework or iEtD ajd2 framework$).mp.
9. strength of recommendation.mp
10. ((grade or grading) adj2 (evidence or recommendation*)).ab,ti.
11. 6 or 7 or 8 or 9 or 10
12. 5 AND 11
13. limit 15 to yr="2015 -Current"

The reference list of all included documents will be screened for additional studies using citationchaser ^22^ supplemented by hand-searching reference lists where necessary.

The following databases, repositories, and websites will be filtered and screened for guideline EtDs, following the approach adopted in a scoping review of public health guidelines ^23^:

- the GIN international guideline library and registry of guidelines in development https://guidelines.ebmportal.com/
- BIGG international database of GRADE guidelines https://sites.bvsalud.org/bigg/en/biblio/
- Epistemonikos GRADE guidelines repository https://www.epistemonikos.org/en/groups/grade_guideline
- GRADEpro Database of GRADE EtD’s and Guidelines https://gradepro.org/guidelines/
- MAGICapp ( https://app.magicapp.org/#/guidelines

Given the recency of the use of GRADE EtD (published in 2016), we will limit the search to an eight year span (2015–2023). The guideline authors will not be contacted; only information provided in the guidelines will be used. It is proposed to include a random sample of 200 (using a random number generator) of identified published EtDs, due to the feasibility of a very large volume being identified for inclusion.

### Selection and screening

Following the search, all identified citations will be collated and imported into Endnote X8. After deduplication, citations will be uploaded into Covidence. The title and abstracts will be screened independently by pairs of reviewers (so that each record is screened by at least two people) for assessment against the inclusion criteria for the review. The full text of selected citations will be assessed in detail against the inclusion criteria by two or more independent reviewers (so that each record is screened by at least two people) in Covidence. Reasons for exclusion of full texts will be recorded and reported. Any disagreements between the reviewers at each stage of the screening process will be resolved through discussion, or with an additional reviewer/s.

### Data extraction

Using a standardized extraction form specifically designed for this review, we will conduct a pilot exercise using 10% of the sample (selected based on Covidence display, most relevant) for the data extractors to calibrate and test the review form. Each document will be extracted by pairs of reviewers working independently (so that each document is extracted by at least two people)

For included empirical studies, commentaries, and discussion papers ( Table 3), data will be extracted on:

- study characteristics (e.g., author, publication year, context, geographic setting)
- study design (e.g., quantitative or qualitative) and approach to evaluation (e.g. survey, focus groups, interviews)
- study participants (disciplines, amount)
- description of application of EtD
- evaluation outcome data

**Table 3.** Data extraction template for empirical studies.

Study characteristics				Study design and participants			Description of application of EtD		Evaluation outcome
Author/ Year	Country	Care Setting	Clinical Area	Study Design	Evaluation methods	Study Participants	EtD Distributed before meeting	EtD application	
Free text	Free text	Options: • Primary • Secondary • Tertiary • All • Other • Not applicable	Free text	Options: • Quantitative • Qualitative • Mixed Methods	Options: • Interviews • Focus groups • Survey • Observations • Mixed • Other	Total N: Brief description, e.g., Nurses, Patient Reps, etc.	Options: • Yes • No • Not applicable	Options: • Structured discussion • Unstructured discussion • Not applicable	Free text

For included EtDs ( Table 4), data will be extracted on:

- the characteristics of the original guideline (e.g., authors, topic, setting)
- the structure and application of the EtD
- the type of evidence source/information used within each of the EtD criteria. Specifically, we will be interested in:
  - Was a systematic review conducted?
  - Why types of evidence (RCTs, NRSI, qualitative studies, formal economic evaluations, health utility studies, panel input, expert consensus etc) are used and where are they cited.

**Table 4.** Data extraction template for EtDs

Guideline characteristics				EtD use description			EtD criteria example (extraction will occur for each criteria)			
Title	Organisation	Year of publication	Clinical Area	Location of EtD	Application of EtD	No of criteria included	Problem Priority included	Supporting evidence supplied	Type supporting evidence supplied	GRADE certainty supplied
Free text	Free text	• Free text	Free text	Options: • Appendix • Glossary • Main body • Other	Options: • Full • Partial	Total N:	Options: • Yes • No	Options: • Yes • No	Options: • Peer-Reviewed research • Expert opinion • Public opinion, • Mix of research & opinions • Guideline group opinion	Options: • Yes • No If yes, which GRADE system

## Data analysis and presentation

All included articles will be described in detail in tables and figures and summarised using descriptive statistics. Published EtDs will be analysed using summative content analysis ^24, 25^, aimed at identifying and quantifying which criteria are presented and the type of evidence source/information used.

## Study status

At time of publication of this protocol, database searches have been carried out and title and abstract screening is currently underway.

## Dissemination

We intend to disseminate the results through publication in a peer-reviewed journal and conference presentations. We will present our findings to professionals engaged in the development of clinical guidelines through the networks of the co-author team.

## Discussion

This scoping review aims to identify the key characteristics of how the GRADE EtD is used and identify studies on perception of use by clinical guideline developers. Given its widespread use and endorsement, it is important to evaluate the user experience of the GRADE-EtD framework. Understanding how GDG stakeholders perceive and use the EtD framework will inform how best to provide training and support - an important facilitator of engagement, particularly patient engagement.

## Data Availability

No data are associated with this article.
