# Supplementary material for: Investigating how the GRADE Evidence to Decision (EtD) framework is used in Clinical Guidelines: a scoping review protocol
Source: HRB Open Res. 2023 Sep 13;6:50. [Version 1] doi: 10.12688/hrbopenres.13757.1 (PMC11109711; doi:10.12688/hrbopenres.13757.1)
Supplement: Peer review history

# Reviewer report: Reviewer response for version 1

**DOI:** 10.21956/hrbopenres.15049.r38906

**Published:** 2024-05-11

**Authors:** Jarrod Dusin

**Affiliations:** 1 The University of Kansas Medical Center, Kansas City, USA

The study aims to understand the application and perception of the GRADE EtD framework by those involved in developing clinical guidelines. Guidelines are important for informing healthcare decisions and improving patient outcomes. The GRADE EtD framework provides a systematic and transparent approach to guideline recommendations. Understanding how guideline groups utilize the GRADE EtD framework could optimize its effectiveness and provide necessary training and support. This paper will provide a much-needed understanding of how the EtD process is used. 

**Major Points Feedback:**

- **Methods:** The methods section is detailed and outlines a clear process for conducting the review. 

- Specify the criteria for inclusion and exclusion of studies in the review. 
- Discuss how disagreements between the two researchers during screening, selection, and extraction will be resolved.
- **Expand on Data Analysis:** 
- The data analysis section could benefit from a more detailed explanation of how the descriptive statistics and content analysis will be conducted. This could include information on the specific statistical tests that will be used and how the results will be interpreted.

**Minor Points Feedback:**

- Please mention any potential limitations or challenges you anticipate in conducting this review.

Is the study design appropriate for the research question?

Yes

Is the rationale for, and objectives of, the study clearly described?

Yes

Are sufficient details of the methods provided to allow replication by others?

Yes

Are the datasets clearly presented in a useable and accessible format?

Yes

Reviewer Expertise:

My work involves the development of guidelines and clinical pathways. I have used both GRADE and EtD in my work.

I confirm that I have read this submission and believe that I have an appropriate level of expertise to confirm that it is of an acceptable scientific standard.

# Reviewer report: Reviewer response for version 1

**DOI:** 10.21956/hrbopenres.15049.r37720

**Published:** 2024-01-18

**Authors:** Chris Carmona

**Affiliations:** 1 National Institute for Health and Care Excellence, Manchester, UK; 2 University of Sheffield, Sheffield, UK

This well designed protocol outlines a scoping review to explore how the GRADE EtD framework is used by guideline developers. It will do this in two ways, it will summarise the current published and grey literature about experiences of using the EtD framework and will also sample 200 EtDs that have been used by guideline committees. The literature will be summarised using descriptive statistics and in depth evidence tables and figures. The EtD content will be analysed using a form of content analysis.

 The proposal is well written and clear and will ensure that a transparent process is followed. I note that some large guideline producing agencies (for example NICE in the UK) do not use EtD frameworks for their guideline recommendations and this research will not explore why it isnt used by some agencies, however I suspect that is a separate research project.

Is the study design appropriate for the research question?

Yes

Is the rationale for, and objectives of, the study clearly described?

Yes

Are sufficient details of the methods provided to allow replication by others?

Yes

Are the datasets clearly presented in a useable and accessible format?

Not applicable

Reviewer Expertise:

Health guideline methods, systematic reviewing, qualitative evidence synthesis

I confirm that I have read this submission and believe that I have an appropriate level of expertise to confirm that it is of an acceptable scientific standard.
